# Mechanically-Durable Antireflective Subwavelength Nanoholes on Glass Surfaces Using Lithography-Free Fabrication

**DOI:** 10.1021/acsami.3c15391

**Published:** 2024-04-05

**Authors:** Iliyan Karadzhov, Bruno Paulillo, Juan Rombaut, Karl W. Koch, Prantik Mazumder, Valerio Pruneri

**Affiliations:** † ICFO-Institut de Ciéncies Fotóniques, Castelldefels, 08860 Barcelona, Spain; ‡ Corning Research and Development Corporation, Sullivan Park, Corning, New York 14831, United States; § ICREA-Institució Catalana de Recerca i Estudis Avançats, 08010 Barcelona, Spain

**Keywords:** antireflection, glass nanoholes, metal dewetting, nanofabrication, durable nanostructures

## Abstract

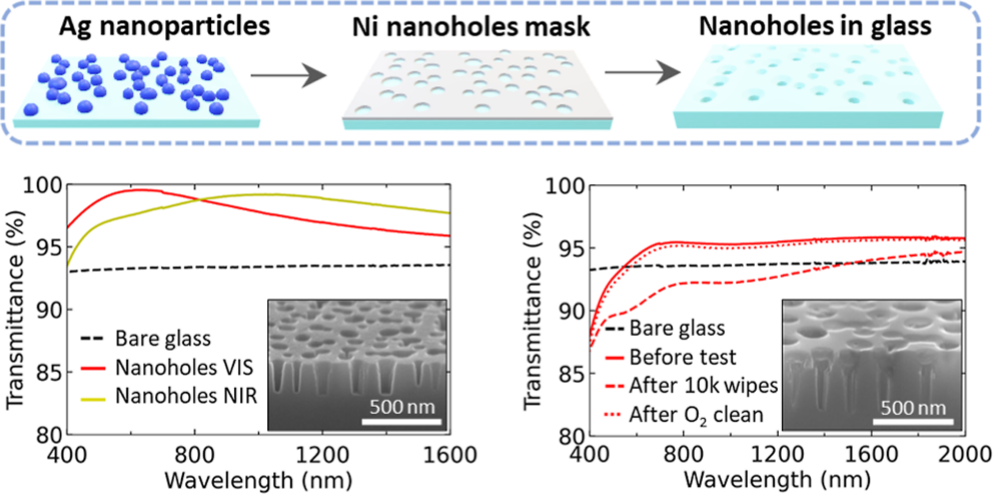

Traditional multilayer antireflection (AR) surfaces are of significant importance for numerous applications, such as laser optics, camera lenses, and eyeglasses. Recently, technological advances in the fabrication of biomimetic AR surfaces capable of delivering broadband omnidirectional high transparency combined with self-cleaning properties have opened an alternative route toward realization of multifunctional surfaces which would be beneficial for touchscreen displays or solar harvesting devices. However, achieving the desired surface properties often requires sophisticated lithography fabrication methods consisting of multiple steps. In the present work, we show the design and implementation of mechanically robust AR surfaces fabricated by a lithography-free process using thermally dewetted silver as an etching mask. Both-sided nanohole (NH) surfaces exhibit transmittance above 99% in the visible or the near-infrared ranges combined with improved angular response at an angle of incidence of up to θ_i_ = 60°. Additionally, the NHs demonstrate excellent mechanical resilience against repeated abrasion with cheesecloth due to favorable redistribution of the shearing mechanical forces, making them a viable option for touchscreen display applications.

## Introduction

Efficient operation of optoelectronic devices such as photovoltaic cells, light emitting diodes, display screens, or imaging systems benefits from reduction in optical reflection that occurs at two interfaces with different refractive indices.^1−3^ For example, a typical air-glass interface contributes to about 4% reflection loss per surface at a normal angle of incidence (AOI). A common mitigation approach entails antireflection (AR) coating that consists of several dielectric layers with optimized thicknesses and refractive indices such that the net destructive interference is achieved for the back reflected beam.^4−6^ One of the most common designs involves SiO_2_ and TiO_2_ continuous films as the low and high refractive index materials.^7^ Current state-of-the-art AR coating can achieve low values of reflection for a specific wavelength range, and when combined with high scratch resistance properties, they are a viable option for consumer touchscreen devices.^8^ However, these designs could still suffer from limited spectral and angular operation range, thermal expansion mismatch, and limited laser-induced damage threshold.^9^

Another approach to suppress interface reflection involves modifying the texture of the surfaces. Originally proposed in 1879 by Rayleigh,^10^ continuous and gradual change of the refractive index between air and the substrate, achieved through nanostructured surfaces that inhibit reflections, has been studied both theoretically^11,12^ and experimentally.^13−15^ Adjusting the volume fraction of air-dielectric (porosity) leads to a smooth transition of the refractive index at the interface and results in a broadband and omnidirectional AR effect.

Nature has developed antireflective surfaces in the evolving species. Inspired by the periodic hexagonal nanosized corneal nipple arrays in the compound eyes of moths,^16,17^ the wings of butterflies,^18,19^ or cicadas,^20,21^ biomimetic subwavelength structures with outstanding spectral and angular antireflective properties have been fabricated. During the past decade, tremendous research efforts have been dedicated to both design and fabrication of AR nanostructured surfaces with various geometrical shapes and arrangements such as nanopillars,^22−31^ nanocones,^32−34^ nanoholes (NHs),^35−39^ and hybrid designs combining nanostructures on top of multilayer thin film stacks.^40−42^ In addition to the improved optical properties, nanostructured surfaces can change the wetting of the surfaces,^25,28,30,32,36,37,42,43^ therefore providing self-cleaning and antifogging effects valuable in touchscreen displays, automotive windows, outdoor solar panels, to name a few. Monolithic substrates (subtractive as opposed to additive process) can also be advantageous in high-power laser applications by raising the laser-induced damage threshold values to these of the bulk material.^29,44^

NH arrays have been reported to have better mechanical resistance against the lateral force applied at the top of the structure when compared to a similar nanopillar structure.^37,43^ Due to their geometry, holes also perform better than pillars in repeated contamination experiments^38^ or abrasion tests.^43,45^ A crucial requirement for nanotextured surfaces to be implemented in real applications, however, is the necessity to be fabricated by a simple low-cost method capable of processing large area substrates. Many of the aforementioned studies are based on expensive and low throughput methods such as e-beam, laser interference,^23,24,32^ or colloidal lithography.^22,25,26,29,33,37,38^ Recently, metal nanoparticles as etch masks obtained through thermal dewetting have gained interest due to their simplicity in preparation and ability to create structures down to few tens of nanometers over large area substrates.^30,42,45−48^ The desired lateral spacing and dimensions of the nanostructures are tuned by the initial thickness of the metal film and the annealing process parameters.^46^ Random nanoparticle arrays of gold,^49^ silver,^39^ and copper^30,42,46,48^ have previously been used as the template that is transferred to the substrate by the dry etch process.

In the present work, we demonstrate a scalable lithography-free method to fabricate mechanically robust AR surfaces based on random NH structures that exhibit broadband transmittance, where the position of the maxima can be adjusted by changing the process parameters. Our approach utilizes three main steps: first, thermally dewetted silver nanoparticles (AgNPs) on a fused silica (FS) glass substrate play the role of an initial mask that defines the in-plane dimensions. Second, we invert the AgNPs mask to an NHs etching mask by depositing a nickel (Ni) thin film on top of the AgNPs and subsequent wet etching of them. Last, the Ni NHs mask is used in a dry etching process to carve nanocavities with different depths into the glass surface. We show that NHs on both sides of the glass bring the peak substrate transmittance at normal incidence above 99% in the visible (VIS) and near-infrared (NIR) regions by adjusting the fabrication parameters. The nanostructures show two times lower mean reflectance in the VIS or NIR spectral range compared to a bare FS glass for an incident angle of θ_i_ = 60°. Additionally, the mechanical robustness of the NHs was evaluated by using an abrasion test that emulates the cleaning procedure of a screen by a cloth. No physical damage was detected, thus opening a door to potential application of the nanostructures in display and touchscreen devices.

## Results and Discussion

### NH Morphology

**Figure 1:**
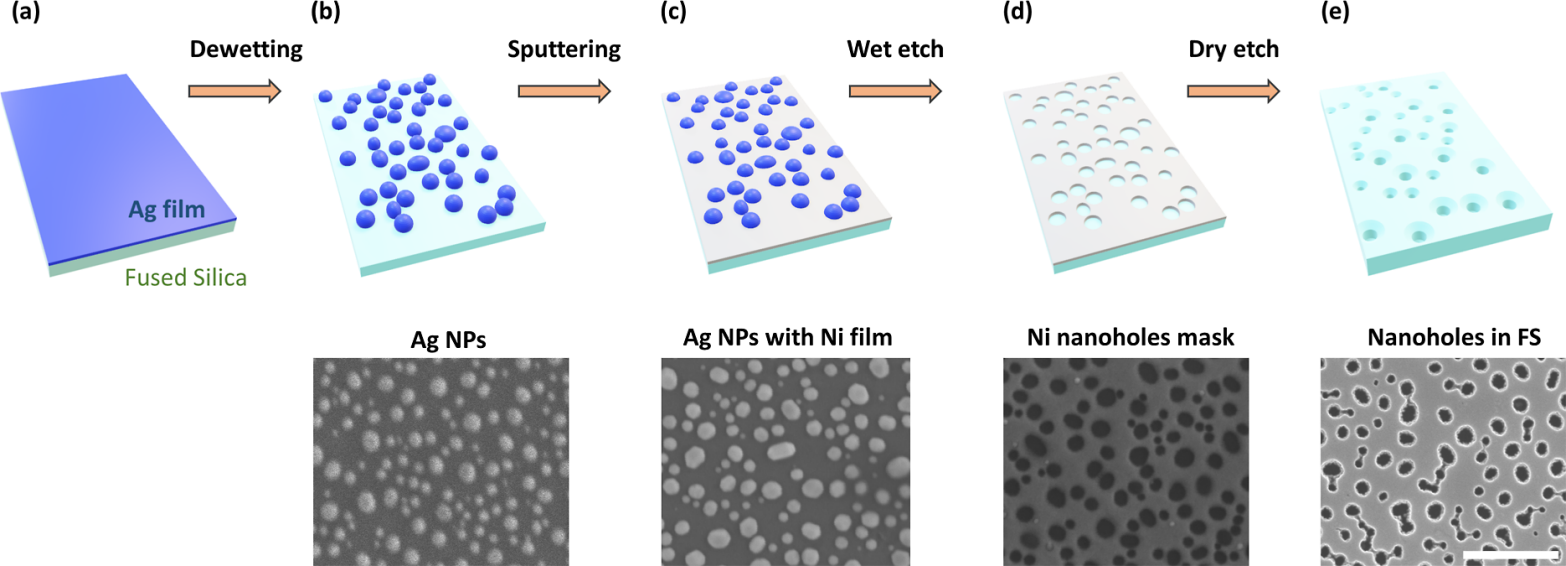
Schematics of the process flow toward fabrication of subwavelength NH structures in glass. (a) Ultrathin silver (Ag) film is sputtered on the FS substrate followed by (b) formation of AgNPs via thermal dewetting. (c) Ni film is deposited on top of them. The partially covered AgNPs are chemically etched (d) and the Ni NHs mask is used in RIE to create the NHs in the glass substrate (e). At each step, representative top view SEM images are provided (scale bar = 500 nm for each one).

The fabrication sequence used to create antireflective holey surfaces in FS glass is presented in Figure 1 and consists of five main steps (see Methods for details). Deposition of a thin metal film followed by formation of nanoislands by solid-state dewetting (Figure 1a,b) is an already established and straightforward method to pattern nanostructures over large glass areas for various applications.^42,45−47,50^ In our proposed method, we transform the dewetted AgNP mask into another etching metal mask, in our case Ni, that has an inverse pattern (Figure 1c,d). Ag was chosen as the metal to define the morphology of the nanostructures due to its ability to dewet readily and form round-shaped nanoislands on glass surfaces^50^ as well as its relatively easy removal with the ammonium persulfate. The initial metal thickness along with the temperature and duration of the dewetting defines the size, shape, separation, and density of the formed AgNPs (see Figure S1 in the Supporting Information). Increasing the metal thickness while keeping other parameters constant results in both larger nanoparticles and interspacing, together with lower densities, as evident in Table S1. An important detail for an easy and successful wet etching step is that the nanoparticle contact angles need to be larger than 90°. This way, the deposited Ni layer covers the top of the AgNPs, but a small area at the bottom of the nanoparticles remains uncovered; thus, the silver etchant can effectively dissolve the AgNPs. The successful wet etching process and the maximum depth of holes that can be achieved both depend strongly on the Ni thickness. We chose nickel because of its chemical inertness to the Ag etchant and its high resilience and selectivity in the RIE process (Figure 1e). Ni films with thicknesses in the range of 15–35 nm were sputtered on top of the samples. The optimal thickness of the Ni film for a given AgNP pattern must be large enough to ensure that sufficiently long dry etching times are needed to reach the desired depth of the NHs. However, it is vital that the AgNP are not completely covered by the Ni film in order to maintain the feasibility of the wet etching process.

**Figure 2:**
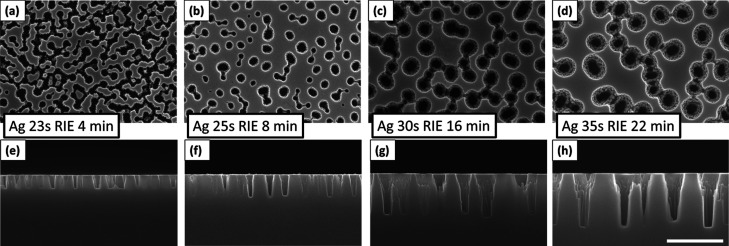
Morphology of the NH structures. (a–d) Top-view SEM images showing that increasing the thickness of the initial Ag film (given as time in seconds) leads to increase in the average diameter of the final NHs. Deposition times of 23, 25, 30, and 35 s are used to define the top diameter of the surfaces. (e–h) Corresponding cross-sectional SEM images of the samples from the top panel where RIE times of 4, 8, 16, and 22 min determine the depths of the nanocavities to be 116, 206, 365, and 470 nm, respectively. All images have the same scale bar = 500 nm.

In Figure 2, top view scanning electron microscope (SEM) images of four different NH structures [top panels (a–d)] are presented along with their cross-sectional scans [bottom panels (e–h)]. Evidently, an increase in the thickness of the initial Ag film (thickness is proportional to deposition time) changes the final arrangement of the holes, i.e., air openings and their separation go up expectedly as it is defined by the previously formed nanoparticles. Longer etching times correspond to deeper NHs as seen from the cross-section SEM images.

However, it is worth noting that the top diameter of all fabricated NHs is consistently larger than the original starting diameter of the AgNPs (see Figure S1 and Table S1 in the Supporting Information). The shape of the NHs is influenced by the thickness of the Ni layer before the dry etch process. It is thinnest at the edge of each hole due to the shadow effect of the nanospheres and grows thicker as it is further from the edges. A thinner layer means that it will be etched away faster around the edge of the holes, which, in turn, when combined with the dynamics and redeposition of RIE, likely causes the holes to have an inverted truncated cone shape (wider top and narrower bottom).

### Optical Performance of NHs in VIS

**Figure 3:**
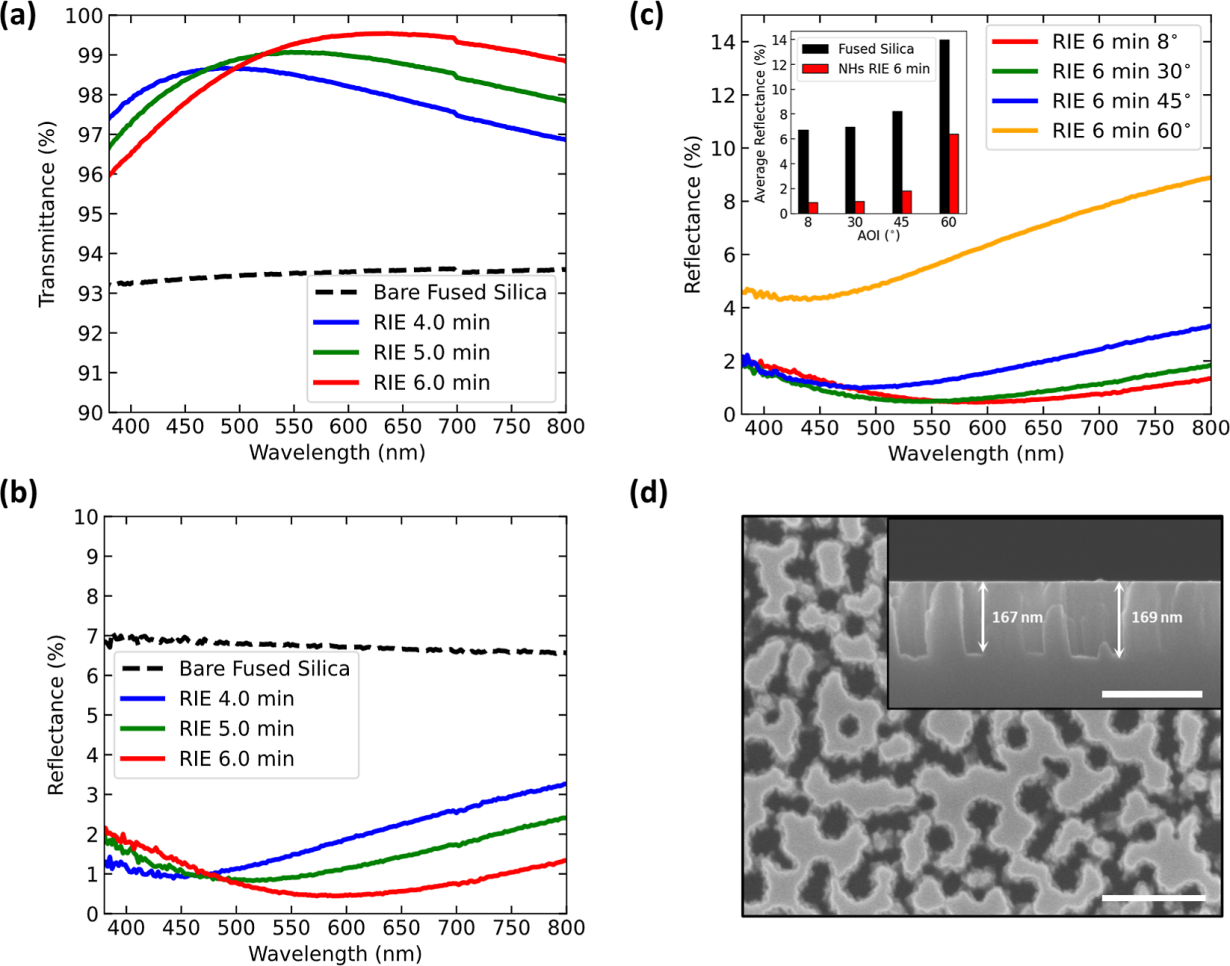
Antireflective properties of double-sided NHs optimized for the VIS range (λ = 400–800 nm). Measured direct (a) transmittance *T*(λ) and (b) reflectance *R*(λ) at normal incidence for three different nanostructures obtained through dry etching times of 4, 5, and 6 min on both sides of the glass. (c) Angular reflectance spectra of sample RIE 6 min at angles of incidence of 8, 30, 45, and 60°. The inset compares the average reflectance in λ = 400–800 nm between the nanostructured RIE 6 min sample and the flat FS surface. (d) Top-view and cross-sectional (inset) SEM images of the sample RIE 6 min (the scale bar in both is 250 nm).

The characteristics of double-sided NH structures with optimized parameters for maximum optical performance in the visible wavelength region (400–800 nm) are presented in Figure 3. The expected optical behavior of the NHs (see Figures S2–S4 in the Supporting Information) was modeled with commercial finite element method software COMSOL Multiphysics (see Methods for details) and used as a design guide in the fabrication process. The dry etching rate of FS glass was about 23 nm/min estimated by cross-sectional SEM measurements (see Figure S5a in the Supporting Information). The transmittance and reflectance spectra in Figure 3a,b of three samples with increasing RIE times at a normal incident angle demonstrate a significant AR effect compared to the flat glass surface. Digital images of the unstructured and nanostructured FS glass slides are provided in Figure S6a in the Supporting Information. As anticipated from the numerical study, NHs, with depths of around 150 nm and nearly vertical sidewalls, provide a maximal AR effect in the VIS. The relation between NH depths (RIE times) and the experimentally determined average transmittance and reflectance presented in Figure S5b corroborates that the optimal etch time is between 5 and 7 min. In particular, the sample RIE 6 min with a measured NH depth of ≈166 nm reaches a maximum in transmittance *T*_max_ = 99.5% at λ = 633 nm and an average transmittance above 99% for a wavelength span of over 300 nm. The sum of measured reflectance and transmittance at λ = 633 nm is close to 100%, indicating negligible scattering of light at that wavelength. Note that *T* and *R* are direct (ballistic) values.

A key optical parameter of nanostructured surfaces relevant for display applications is the induced light scattering when the dimensions of the features are within the order of the incident wavelengths. Haze measurements of the presented three nanostructures resulted in 0.10, 0.17, and 0.30% averaged haze in the visible range for etching times of 4, 5, and 6 min, respectively. Results indicate an increase in scattering with the increasing vertical dimensions of the holes, however, low enough (<0.5%) for display applications.

Angular transmittance and reflectance spectra were acquired for the sample RIE 6 min at angles of incidence (AOI) of 8, 30, 45 and 60°, as shown in Figure 3c. The data are an averaged sum of s- and p-polarization measurements acquired consecutively. The minima of the spectra shift to shorter wavelengths as AOIs increase, still, remaining well below the reflectance of the flat reference surface. The inset in Figure 3c compares the mean reflectance values of sample RIE 6 min and the unstructured flat FS surface in the 400–800 nm interval. At incident angles of 8, 30, 45, and 60°, the reflectance of the NHs is *R*_holes_ = 0.86, 0.95, 1.81, and 6.36%, respectively, while the reflectance of flat glass is *R*_flat_ = 6.71, 6.93, 8.21, and 13.94%. Although the increase of reflectance at larger incident angles is expected for both flat and patterned surfaces, this result indicates the improved omnidirectional AR properties of our NH surfaces and the persistent better performance over the nonpatterned glass surface. This trend is further confirmed by comparison of the visual appearance between bare glass and two-sided NH structured glass when a fluorescent lamp illuminates the samples at oblique angles, as presented in Figure S6c (top and middle row).

### Optical Performance of NHs in NIR

**Figure 4:**
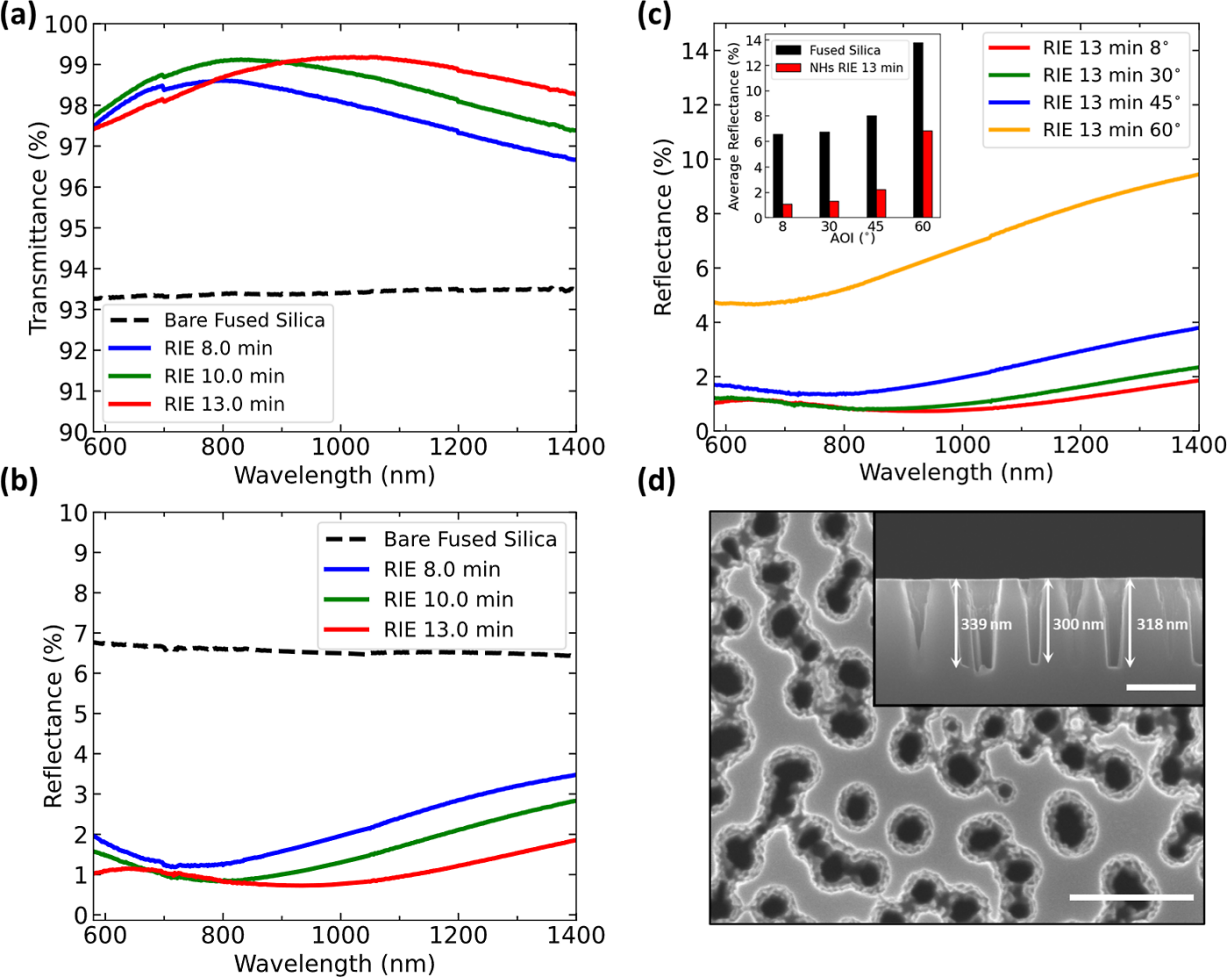
Antireflective properties of double-sided NHs optimized for the NIR range (λ = 600–1400 nm). Measured direct (a) transmittance *T*(λ) and (b) reflectance *R*(λ) at normal incidence for three different nanostructures fabricated after the dry etching process of 8, 10, and 13 min on both sides. (c) Angular reflectance spectra of sample RIE 13 min at angles of incidence of 8, 30, 45, and 60°. The inset compares the average reflectance in λ = 600–1400 nm range between the nanostructured RIE 13 min sample and the flat FS surface. (d) Top-view and cross-sectional (inset) SEM images of the sample RIE 13 min (the scale bar in both is 250 nm).

To further explore the versatile nature of our holey structures in terms of AR properties, we fabricated NHs on both surfaces of the glass substrates with 25 s deposition time of the defining Ag thin film. We were guided by the numerical calculations that suggested depths of 300 nm or more will reduce the substrate’s one-side reflectance below 1% in the NIR range given that the NH top diameter is at least 100 nm and its slope ratio (SR = *D*_bottom_/*D*_top_) is closest to zero (see Figure S3a,b in the Supporting Information). The transmittance and reflectance of the structures in the NIR (λ = 600–1400 nm) region at the normal incident angle are shown in Figure 4a,b. As the hole depths increase, the mean reflectance in the NIR decreases and approaches 1% for sample RIE 13 min, as seen in Figure S5c. The visual appearance of the fabricated samples is depicted in Figure S6b in the Supporting Information. By increasing the RIE times, the spectral position of maximum transmittance could be tuned. The sample RIE 13 min has transmittance *T* = 99.16% and reflectance *R* = 0.86% at λ = 1064 nm with the sum of both *R* + *T* ≈ 100%. Assuming that the FS glass absorption in this wavelength range is negligible, this means that there are no scattering losses at this operational wavelength. Specifically, the scattering losses of sample RIE 8 min and RIE 13 min become ≈0% for λ > 900 nm and λ > 1050 nm, respectively. The results are in agreement with the simulated reflectance spectra for samples RIE 8 and 13 min, as shown in Figure S3c, where the offset of ≈0.6% may be related to the variance in the geometric dimensions due to the random nature of our NHs.

Figure 4c displays the measured reflectance of the RIE 13 min sample at oblique incident angles of 8, 30, 45, and 60° together with the mean values of the nonstructured and structured glass surface in the 600–1400 nm range (in the inset). Digital images of this sample are provided in the bottom row of Figure S6c in the Supporting Information. The measured reflectances of the NHs and the bare glass are *R*_holes_ = 1.07, 1.29, 2.22, and 6.82% and *R*_flat_ = 6.55, 6.75, 8.03, and 13.77%, respectively. The NHs demonstrate a substantial improvement in the angular reflectance over the reference flat glass due to their subwavelength nature and the induced gradual change of the effective refractive index given by the volume ratio of air/glass. Figure 4d shows the top-view and cross-sectional SEM images of sample RIE 13 min with an average top diameter of ≈103 ± 20 nm and a depth of ≈310 nm. This depth is roughly three times lower than the wavelength where maximum performance occurs (λ = 1022 nm) and is in agreement with the condition of the nonscattering surface.^27^

### Mechanical Durability of NHs

**Figure 5:**
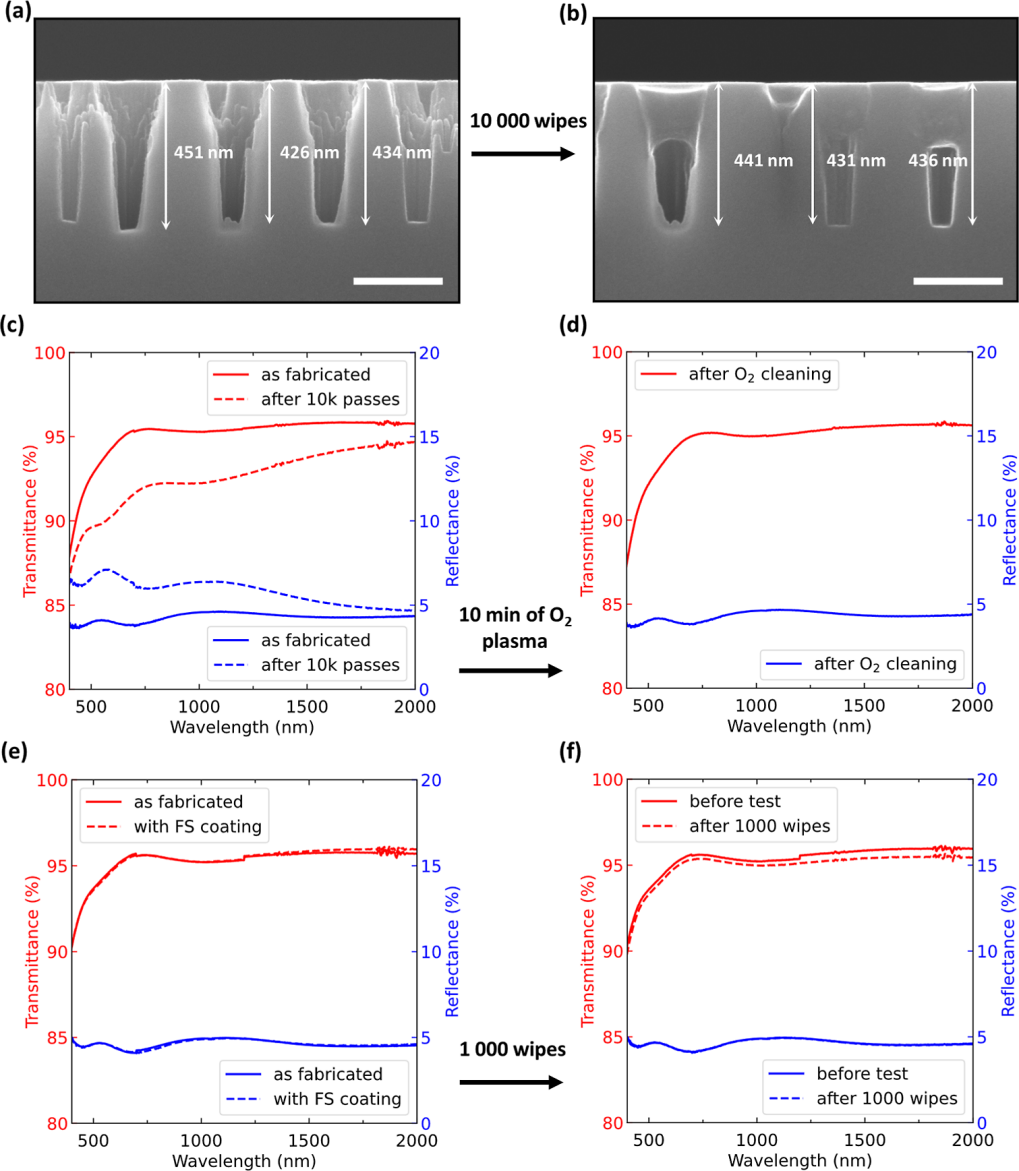
Mechanical durability test results of the proposed NH structure after 10,000 passes of a cheesecloth with a crockmeter applying a constant load of 7.35 N over 3.14 cm^2^ contact area. Cross-sectional SEM images before (a) and after (b) the abrasion show that the holes are partially filled with residual cloth material (the scale bar in both is 250 nm). (c) Transmittance (red, left axis) and reflectance (blue, right axis) of the sample before (solid lines) and after the test (dashed lines). (d) After oxygen plasma cleaning, the transmittance and reflectance spectra return to values very close to those before the test (after fabrication). (e) Transmittance (red, left axis) and reflectance (blue, right axis) of another NH structure sample with slightly shallower depths before (solid lines) and after coating it with a fluorosilane coating (dashed lines). (f) After the crockmeter test consisting of 1000 double passes, they essentially preserve their optical response.

The mechanical robustness of all types of nanotextured surfaces is paramount when implementing surfaces into commercial products. The higher and the thinner the nanostructures, the larger the tangential and normal stresses due to physical contacts with the surface.^37^ For touchscreen displays, one relevant reliability test is the crockmeter test.^51^ We chose to perform the test on nanostructures with a 436 ± 8 nm depth after 20 min of dry etch (Figure 5a). After 10,000 single passes of a cheesecloth fabric, SEM inspection revealed that the holes were partially filled with residues from the cheesecloth, as evident in Figure 5b. Correspondingly, the measured transmittance and reflectance of the tested area over a broad spectral range (Figure 5c) convey a decrease in the transparency after the test, with a stronger effect for shorter wavelengths. After applying oxygen plasma cleaning for 10 min, the sample completely restored its initial optical behavior, as shown in Figure 5d, implying that our NHs had remained structurally intact during the abrasion test.

Furthermore, to show that our nanostructured surfaces can readily be applied in consumer touchscreen displays without the need for special cleaning methods, we applied perfluoropolyether with a silane group, referred to as FS coating, which was sprayed onto a NH sample with ≈400 nm hole depth (dry etch of 17 min) and bonded to the glass surface after thermal treatment (see Methods for details). This coating provides two functions. It prevents direct contact between the surface and the abrader and lowers the coefficient of friction leading to less transfer of material from the cloth into the NHs. As shown in Figure 5e, the ultrathin layer of FS coating did not affect the transmittance and reflectance spectra of the tested sample. After the abrasion test consisting of 1000 double passes of cheesecloth fabric with a constant load, the NH structures showed practically no change in their optical properties, as seen in Figure 5f. Therefore, the additional polymer coating serves as a reliable protection against potential contaminants being stuck inside the NHs. Note that the FS coating also made the NH substrates hydrophobic. For example, the tested sample in Figure 5e had a water contact angle of 118.8 ± 2.1° obtained at five different positions across the sample surface.

## Methods

### Fabrication of Nanocavities on Glass Surfaces

High purity FS optically polished glass with a thickness of 1 mm and an area of 4 square in. were used as substrates to pattern the NH structures on both sides. Initially, surfaces were cleaned with acetone, followed by deionized water in an ultrasonic bath, each process lasting for 10 min. Samples were dried by blowing nitrogen. Next, ultrathin Ag films with different thicknesses (between 7 and 12 nm) were deposited by magnetron sputtering (ATC Orion 8, AJA International, Inc.) on the FS substrates. Prior to all depositions, a cleaning step of argon (Ar) plasma [50 W bias power, 40 standard cubic centimeter per minute (sccm) at 5 × 10^–3^ Torr] for 5 min was employed in the main chamber to additionally clean samples. The substrates underwent depositions at room temperature, 75 W of DC-power with 20 sccm of pure Ar flow. The base pressure was 10^–8^ Torr, the working pressure was 2 × 10^–3^ Torr, and the substrate-target distance was 32 cm. The deposition rate of 0.33 nm/s was determined by measuring the thicknesses of sputtered films for various times using an ellipsometer (J. A. Woollam Company). Next, the substrates were subjected to rapid thermal annealing process (TsunamiTM RTP600S) at 750 °C for 135 s resulting in dewetting of the Ag films into round-shaped AgNPs. The initial thickness of the Ag films was varied to control the average size and separation of the formed nanoislands.^46^ In order to prevent the oxidation of films in the oven, a mixture of hydrogen (4%) and nitrogen was blown inside it. After dewetting, Ni films of different thicknesses (15–35 nm) were sputtered on top of the AgNPs using the same chamber conditions as the Ag films. To remove the AgNPs and create the Ni NH mask, samples were immersed in ammonium persulfate (APS-100, Transene Co.) while being placed in an ultrasonic bath for 40 min and subsequently dried with nitrogen. The glass nanocavities were created using a reactive ion etching (RIE) system (Plasmalab System 100, Oxford Instruments) operated at room temperature, 10 mTorr pressure, 300 W RF-power, and 40 sccm Ar/5 sccm CHF_3_ plasma. Controlling the depth of the structures was achieved by adjusting the process duration. After etching, to remove the mask residuals, the substrates were treated with Ni etchant (30 wt % FeCl_3_, Sigma-Aldrich) for several seconds. As the final step, the samples were sonicated with acetone and DI water for 10 min each and treated in a plasma asher (Branson) with a 300 mL/min flow of O_2_ and 550 W power.

### Hydrophobic Fluorosilane Coating

The surface free energy of the samples was modified by applying an alkoxysilane-modified perfluoropolyether. Samples were treated with oxygen plasma (200 W, 1.5 Torr, 3639 ccm) using a Branson plasma chamber for 5 min to remove organics from the surface prior to application of the fluorosilane coating. Daikin Optool UD509 was diluted to 0.6% (v/v) using Novec 3 M 7200 engineering fluid to result in 0.12% final polymer concentration. It was prepared and stored in a humidity-controlled environment (<2% humidity) until deposition. The spray coater used in this experiment was Asymtek SL 940E. Samples were carefully loaded into the tool and sprayed using an atomization pressure of 30 psi and spray raster of 20 in. per second at a 0.7 in. pass width. Samples were cured in a Baxter DX-31 drying oven in ambient air for 30 min at 150 °C. They were then rinsed in a Novec 3 M 7200 engineering fluid bath, using a Branson 5800 sonicator for 10 min. Once sonication was complete, they were removed from the bath and air-dried.

### Optical, Morphological, Wetting, and Mechanical Characterization

The directional transmittance and reflectance spectra were acquired in the wavelength range of 380–2500 nm by using a UV–vis–NIR spectrophotometer (Cary 7000 UMS, Agilent) that enabled changing the AOI between 0 and 75° along with changing the beam polarization. All reflectance and transmittance spectra presented here are an averaged sum of s- and p-polarization data, with only the transmittance at normal incidence (AOI = 0°) being recorded for an unpolarized beam. The scattering of the surfaces in the VIS region was quantified by measuring the haze parameter, that is, the amount of incident light diffused by the sample at an angle larger than 2.5° divided by the total light transmitted by the sample, with a hazemeter (Haze-Gard I, BYK Gardner). To determine the scattering losses *S*(λ) in the NIR region, we assumed that for nonabsorbing glass, *S*(λ) ≈ 100 – *R*(λ) – *T*(λ). The morphology of the nanostructured surfaces was examined by a field-emission scanning electron microscope (Hitachi SU-70) operating in a secondary electron imaging mode at 5 kV. The water contact angles were measured using a drop shape analysis system (DSA-100, Krüss). The mechanical durability of the structures was tested using a crockmeter (M238BB, SDL Atlas) with a special cloth used in standard test 8 approved by the American Association of Textile Chemists and Colorists. The abrading surface was 3.14 cm^2^ with a constant applied force of 7.35 N. Each cycle consists of two single passes with a stroke length of 50 mm.

### Modeling of Optical Response

Finite element method commercial software (COMSOL Multiphysics 5.4) was used to perform full-wave electromagnetic simulations (see Figures S2 and S3 in the Supporting Information). The nanostructures were modeled by using periodic boundary conditions and a square lattice with a centered nanocavity. All simulated spectra represent only a one-side response due to the addition of an absorbing boundary condition (perfectly matched layer, PML) introduced at the bottom. The geometric parameters that defined the model and were varied to study the optical behavior were the period (*P*), the period ratio (defined as PR = *D*_top_/*P*), the slope ratio (defined as SR = *D*_bottom_/*D*_top_), and the depth *D*_hole_ of the NH (see Figure S2a). The FS glass dispersion relation was taken from the Malitson model.^52^ Based on statistical analysis of the SEM data, the period of simulated NHs was fixed to *P* = 100 nm and *P* = 130 nm for structures performing best in the VIS and NIR wavelength range, respectively. Note that the experimental NH structures are random whereas a periodic NH lattice with statistically averaged dimensions (hole diameter, hole depth, and hole number density) was used for simulations. Hence, the numerical study reported in the Supporting Information is useful as a design guide for identifying how the geometrical parameters affect the reflectance evolution in order to optimize the nanofabrication. On the other hand, simulations only give a qualitative agreement between the simulated and experimental reflectance data that can be expected (see, for example, Figure S3c in the Supporting Information).

## Conclusions

In this paper, we demonstrated a facile fabrication of nanostructured glass surfaces, namely, random NHs, that possess excellent antireflection properties due to the gradual change of the effective refractive index. The transmittance of the double-side NH-structured surfaces reached >99% and can be tuned for different spectral ranges (VIS, NIR) depending on the application. The NH surfaces have low scattering, high broadband transparency and, improved angular response, and they show mechanical robustness proved by abrasion tests. Our approach relies on inexpensive and scalable lithography-free techniques, such as thermal dewetting and dry etching, to generate and control the geometry of the NHs. We believe that all these features coupled with the proposed feasible and scalable manufacturing process are attractive for implementation of the NH surfaces in various optoelectronic devices and in particular as cover glass in touchscreen displays.

## Supporting Information Available

SEM images of dewetted AgNPs and statistics of the geometric parameters; expected optical behavior of nanocavities using the finite element method simulations by varying the defining nanocone parameters; calculated change in the mean transmittance and reflectance of the NHs as a function of their top diameter; experimental relationship between NH depths and mean transmittance/reflectance; and digital photos of the bare glass and the nanostructured surfaces under different AOIs (PDF)
