# Supplementary material for: Mechanically-Durable Antireflective Subwavelength Nanoholes on Glass Surfaces Using Lithography-Free Fabrication
Source: ACS Appl Mater Interfaces. 2024 Apr 5;16(15):19672–80. doi: 10.1021/acsami.3c15391 (PMC11040582; doi:10.1021/acsami.3c15391)
Supplement: Supplementary file 1 — am3c15391_si_001.pdf [file am3c15391_si_001.pdf]

Supporting Information
Mechanically-Durable Antireflective Subwavelength Nanoholes on Glass Surfaces Using Lithography-Free Fabrication

Iliyan Karadzhov,[*,†] Bruno Paulillo,[†] Juan Rombaut,[†] Karl W. Koch,[‡] Prantik Mazumder,[‡] and Valerio Pruneri[*,†,¶]

†*ICFO-Institut de Ciéncies Fotóniques, Castelldefels, 08860 Barcelona, Spain*

‡*Corning Research and Development Corporation, Sullivan Park, Corning, New York 14831, United States*

¶*ICREA-Institució Catalana de Recerca i Estudis Avançats, 08010 Barcelona, Spain*

E-mail: iliyan.karadzhov@icfo.eu; valerio.pruneri@icfo.eu

1. Dewetted silver nanoparticles (AgNPs) on glass.

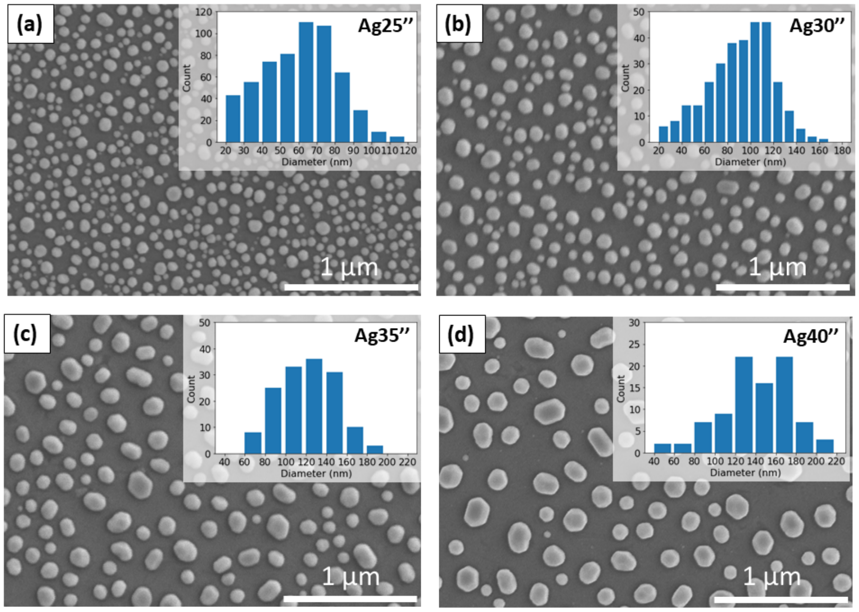

Figure S1: Top view SEM images and distributions of dewetted silver nanoparticles (AgNPs) on fused silica. The deposition times were 25 (a), 30 (b), 35 (c), and 40 sec (d). Insets represent the histogram of nanoparticle diameters obtained through image analysis with the ImageJ tool.

Table S1: Summary of the parameters that define the initial morphology of the NHs (mean diameter, standard deviation, particle density, average spacing and filled area fraction) for increasing initial silver thicknesses.

Deposition time (sec)	$\langle D \rangle \pm$ stdev (nm)	Particle density (μm^{-2})	Average spacing (nm)	Filling fraction (a.u.)
25	65 ± 20	96	102	0.32
30	96 ± 28	37	165	0.27
35	125 ± 27	19	226	0.24
40	147 ± 37	12	287	0.21

Figure S1 and Table S1 summarize the arrangement of the AgNPs on the FS glass surface after the dewetting process. Despite the randomness, SEM measurements across multiple locations on the samples result in very similar morphology parameters, meaning that structures are statistically uniform across the entire sample surface of 4 square inches.

2. Finite element method simulations of nanocavities.

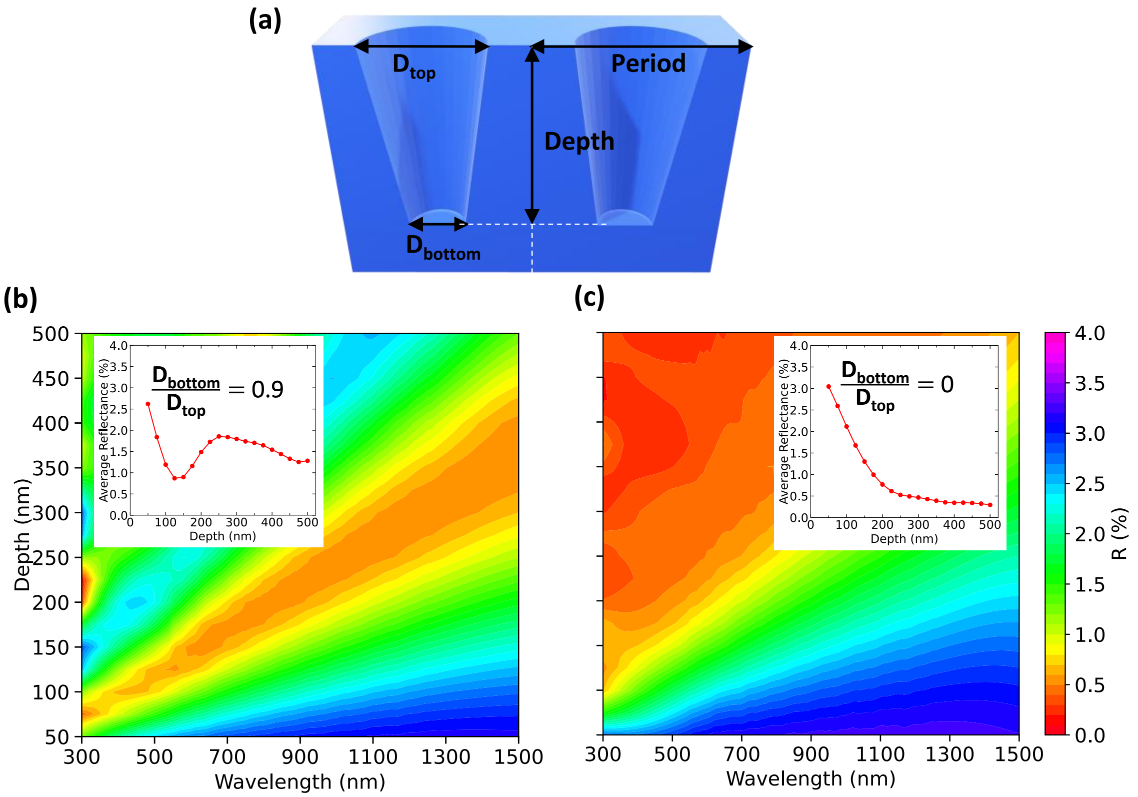

Figure S2: Simulated one-side reflectance R at normal incidence of nanocavities on glass in the 300 - 1500 nm range for fixed period $P = 100\,\text{nm}$ and period ratio $PR = D_{\text{top}}/P = 1.0$. (a) Schematics of the structure and the geometrical parameters that were varied through a parametrical sweep. Contour plot of reflectance as a function of wavelength and NH depth for the two limit cases: in (b) holes are approaching a cylindrical shape ($SR = 0.9$) while the structure in (c) is an inverted cone ($SR = 0$). The mean reflectance in the $400 - 800\,\text{nm}$ range depending on the depth is shown in both insets.

Figure S2 summarizes the expected optical behaviour of the simulated structure using COMSOL Multiphysics software. The nanocavities were modeled using a periodic squared array with period $P = 100\,\text{nm}$ and period ratio $PR = D_{\text{top}}/P = 1.0$ (touching holes) for two limit cases: sidewalls close to vertical with $SR = 0.9$ (Figure S2b) and pointy bottom with $SR = 0$ (Figure S2c), which is essentially an inverted nanocone. The period was chosen to match the average value obtained from the statistical analysis of SEM measurements after the dewetting process. When the slope ratio is approaching one, equivalent to cylindrical

nanohole, an abrupt change of the effective refractive index (RI) n_{eff} along propagation direction occurs. In this case, the reflectance of the nanohole structures can be viewed as equivalent to a single layer coating with thickness d_{etch} and effective RI n_{eff}. The reflectance minima occur at wavelengths satisfying the condition $\lambda_{\min} = d_{\text{etch}}/4n_{\text{eff}}$ (the orange areas in the contour plot in Figure S2b). The calculated mean reflectance in the $400 - 800$ nm range as a function of the depth suggests that NHs with depths between $100 - 150$ nm will have the lowest mean reflectance values. To achieve a flatter optical response across wider wavelength range and reflectance below 0.5%, more gradual change of the effective refractive index is needed. By choosing the slope ratio to be equal to zero (i.e., nanocone), the discontinuity in the effective RI for the interface nanocone-bulk glass can be eliminated. Holes deeper than 250 nm will result in less than 0.5% mean reflectance as shown in Figure S2c. As the NH depth further increases up to 500 nm, the average reflectance tends to decrease slowly and the low-reflectance region is broadened towards longer wavelengths.

Figure S3 shows the reflectance of the NHs with fixed period $P = 130$ nm and slope ratio $SR = D_{\text{bottom}}/D_{\text{top}} = 0$, i.e., inverted nanocones. In Figure S3a the mean reflectance the $300 - 1500$ nm range is plotted as a function of period ratio $PR = D_{\text{top}}/P$ and depth. Structures with small period ratios ($PR = 0.5$) are not able to suppress the mean reflectance even for the deepest holes because the air-glass fraction at the upper interface results in change of the effective RI from $n_{\text{air}} = 1$ to $n_{\text{air-hole}} = 1.27$. In order to obtain the lowest mean reflectance values, high period ratios and depths of at least 300 nm are required. Figure S3b shows the mean reflectance as a function of wavelength and depth when the top diameter is equal to the period of the lattice ($PR = 1.0$). In this case, the $n_{\text{eff}} = 1.10$ at the air-hole interface. The NHs are able to suppress progressively the mean reflectance with increasing the depth and reach below 0.5% values as shown in the inset of Figure S3b. We also compare in Figure S3c the reflectance spectra of two simulated structures with geometric parameters that closely match the experimentally measured dimensions from SEM images. The simulated spectra follow in good agreement the measured ones and the offset can be

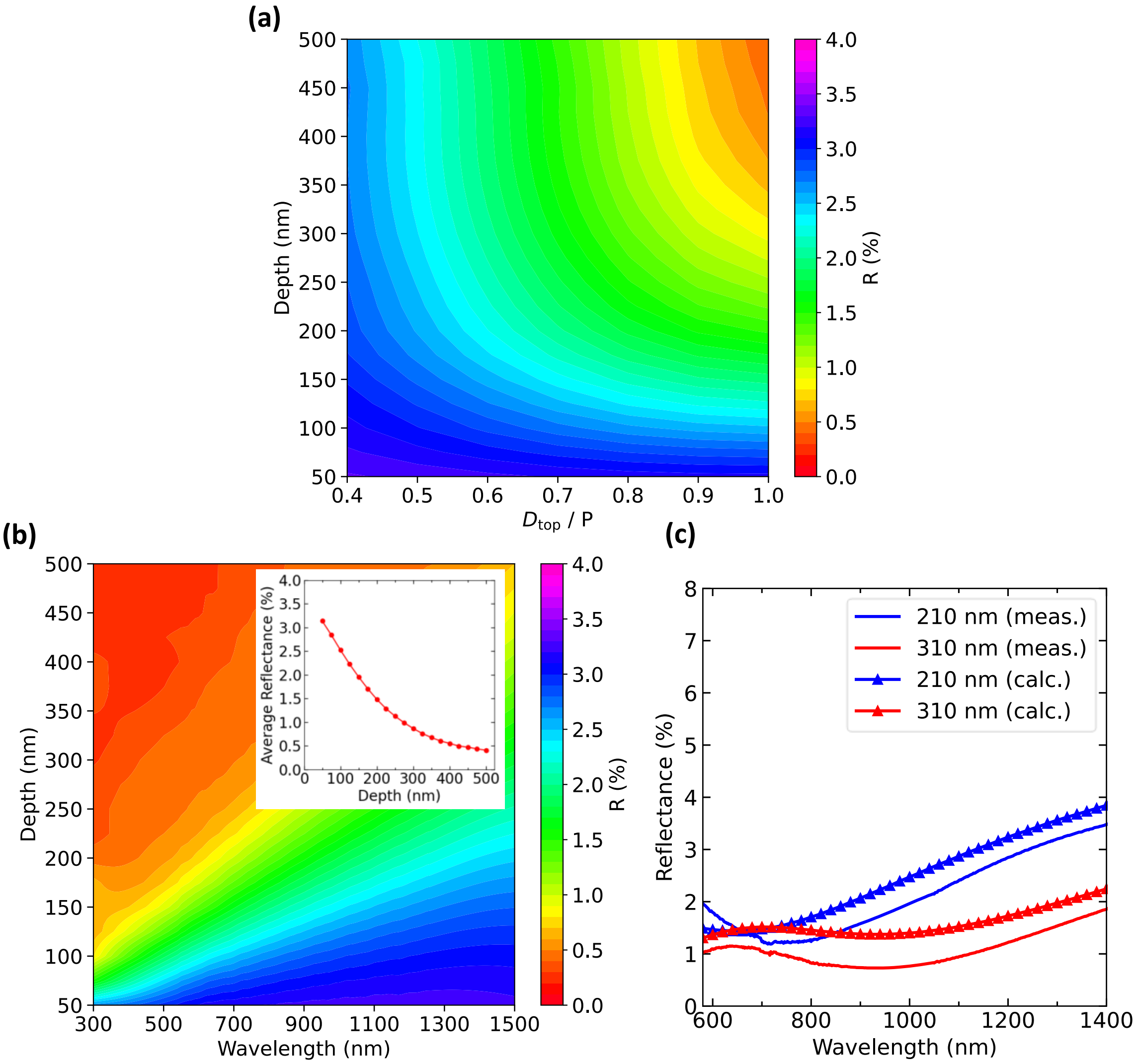

Figure S3: Simulated one-side reflectance R at normal incidence of nanocavities on glass in 300 - 1500 nm range for fixed period $P = 130\,\text{nm}$ and slope ratio $SR = D_{\text{bottom}}/D_{\text{top}} = 0$. (a) Contour plot of the mean reflectance in the $300 - 1500\,\text{nm}$ wavelength range as a function of period ratio (D_{top}/P) and depth. (b) The reflectance as a function of wavelength and depth was evaluated for NHs with top diameter equal to the period of the lattice ($PR = 1.0$). The inset shows the calculated average values of R in the $300 - 1500\,\text{nm}$ interval as a function of depth demonstrating that holes with diameters approaching the period can achieve below 0.5% mean reflectance when sufficiently deep. (c) Comparison between measured (solid) and simulated (triangles) reflectance of samples with depth of cavities of ≈ 210 nm and ≈ 310 nm (samples RIE 8 min and RIE 13 min in Figure 4 in the main text). Parameters of the simulated holes: $P = 130$ nm, $SR = 0.2$, $PR = 0.9$ and $D_{\text{hole}} = 210$ and 310 nm.

attributed to the fact that the fabricated structures have distribution in all four defining geometric parameters. Moreover, as evident in the SEM data, there are single isolated holes and merged holes, the latter are not accounted in the square periodic model we used and might give rise to the overall AR effect.

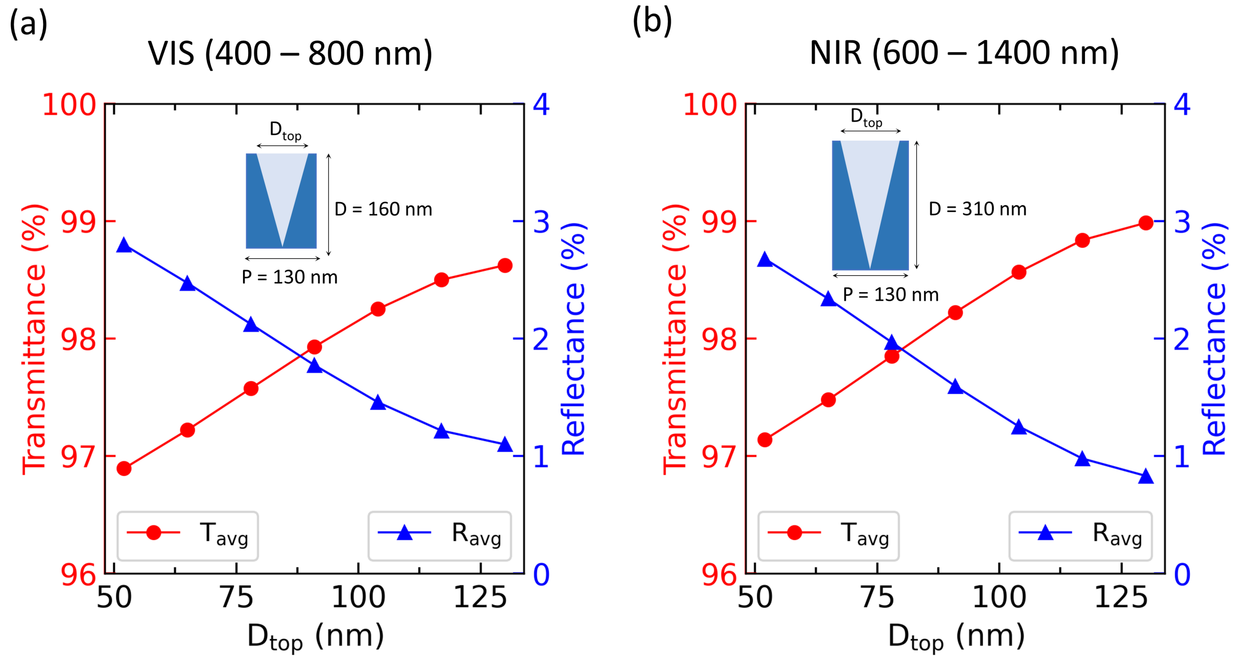

Figure S4: Simulated one-side mean transmittance and reflectance in the VIS (a) and in the NIR (b) of nanoholes as a function of the top diameter D_{top}. The parameters of the simulated structure in (a) are period $P = 130$ nm, slope ratio $SR = D_{\text{bottom}}/D_{\text{top}} = 0$, depth $D = 160$ nm, whereas in (b) the depth $D = 310$ nm.

The calculated transmittance and reflectance of the NHs as a function of the top diameter for two depths, 160 nm and 310 nm, that most closely correspond to the fabricated nanoholes with best optical performance in the VIS and NIR is shown in Figure S4a and Figure S4b, respectively. Larger diameters decrease the overall reflection, however, the minimum achieved reflectance is limited by the remaining glass surface at the interface air-hole when the hole diameters are touching and equal to the period of the cell.

3. Relationship between NH depths and transmittance/reflectance.

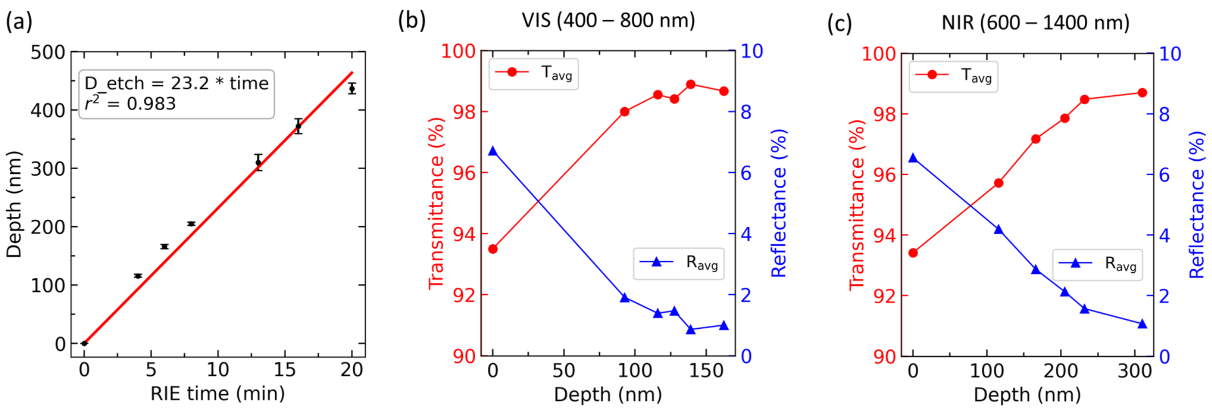

Figure S5: (a) Determination of the dry etch rate of fused silica using a linear fit to six nanohole samples. For each sample, six depths were measured and averaged by cross-section SEM scans. Experimentally measured average transmission (left axis, red) and reflection (right axis, blue) in the VIS range (b) and in the NIR (c) as a function of depth that is determined using the calibration line in (a). The values for bare glass are also plotted.

4. Photographs of bare FS glass and nanoholes (NHs) at different AOIs.

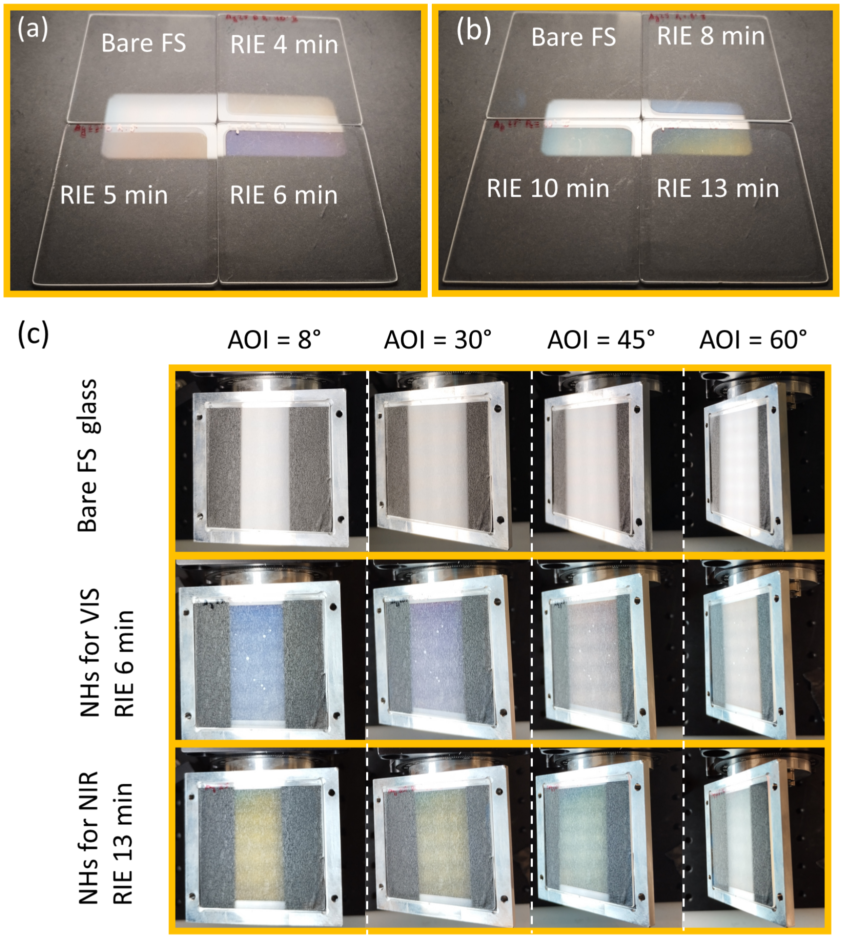

Figure S6: Photographs of the bare FS glass and the three NHs samples optimized for (a) VIS and (b) NIR that correspond to the measured spectra in Figure 3 and Figure 4 in the main text. (c) Photographs of bare FS glass (top row), both-sided NHs sample RIE 6 min (middle row) and both-sided NHs sample RIE 13 min (bottom row) at AOIs $= 8°, 30°, 45°$, and $60°$.
